# Supplementary material for: Associations between fMRI signal amplitude, hemispheric asymmetry, and task performance
Source: Commun Biol. 2025 Oct 8;8:1439. doi: 10.1038/s42003-025-08843-w (PMC12508031; doi:10.1038/s42003-025-08843-w)
Supplement: Supplementary file 1 — Supplementary Information [file 42003_2025_8843_MOESM1_ESM.pdf]

Supplementary Information for

Associations between fMRI Signal Amplitude, Hemispheric Asymmetry, and Task Performance

Dardo Tomasi*[1] and Nora D. Volkow[1]

[1]National Institute on Alcohol Abuse and Alcoholism, Bethesda, MD, 20892, USA

SUPPLEMENTARY RESULTS

Statistical results in CIFTI format

Vertex-wise t-test statistics (t-score maps) with an FDR-threshold p<0.05 for the whole sample (n=989; *BOLD-fMRI_t*qmask.dscalar.nii) and mean values for Discovery (n=504; *BOLD-fMRI1*.dscalar.nii) and Replication (n=485; BOLD-fMRI2*.dscalar.nii) subsamples across 86 contrasts of BOLD signal amplitude and 86 contrasts of asymmetry in CIFTI format are included for detailed review in 42 files (https://figshare.com/s/c31e52f0f2d07bc53e60; DOI: 10.6084/m9.figshare.28204100).

Supplementary Figures

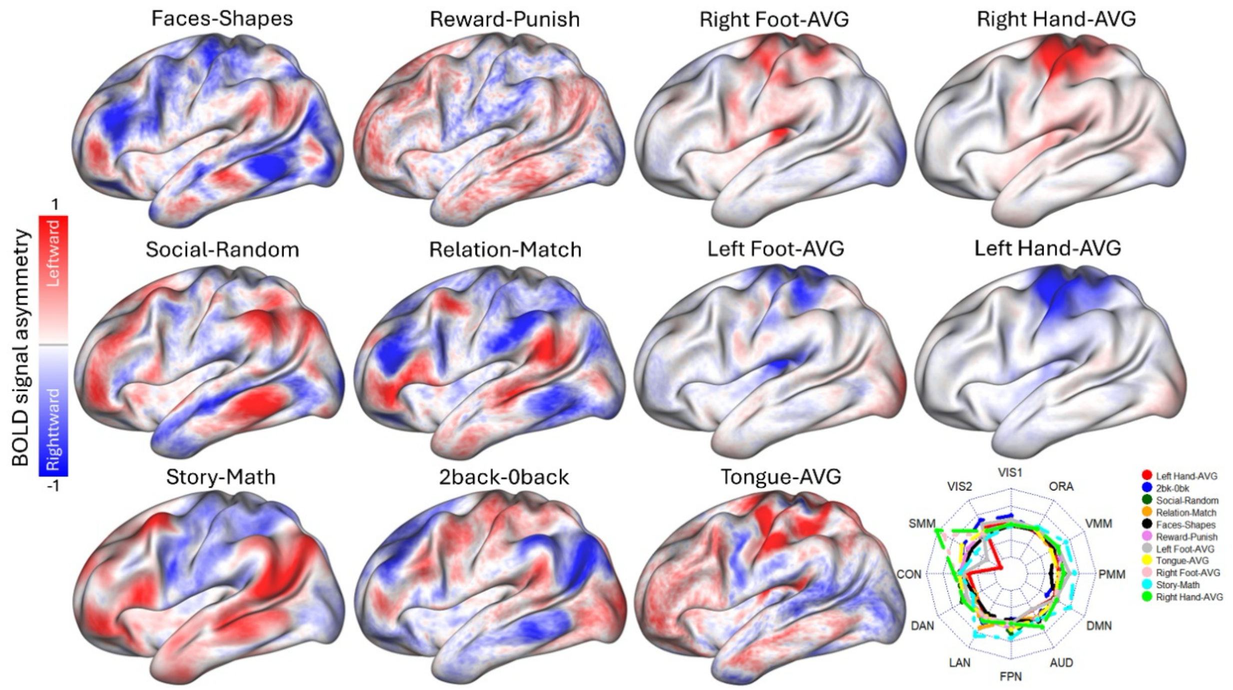

Figure S1. Functional Asymmetry Maps. Vertex-wise asymmetry maps derived from eleven fMRI signal contrasts are displayed: Faces vs. Shapes (Emotion task), Reward vs. Punish (Gambling task), Social vs. Random (Social task), Relation vs. Match (Relational task), Story vs. Math (Language task), 2-back vs. 0-back

(Working Memory task), and Right Foot-Average (AVG), Right Hand-AVG, Left Foot-AVG, Left Hand-AVG, and Tongue-AVG (Motor task). The radar chart (bottom right) summarizes average asymmetry for each contrast across 12 network partitions: Visual (VIS1 and VIS2), Somatomotor (SMM), Cingulo-Opercular (CON), Dorsal Attention (DAN), Language (LAN), Frontoparietal (FPN), Auditory (AUD), Default-Mode (DMN), Posterior (PMM) and Ventral Multimodal (VMM), and Orbito-Affective (ORA) networks. Sample includes 989 healthy young adults. One-sample t-test. P<0.05, FDR-corrected for multiple comparisons. Radar chart inner and outer limits are set at -0.3 and 0.3, respectively.

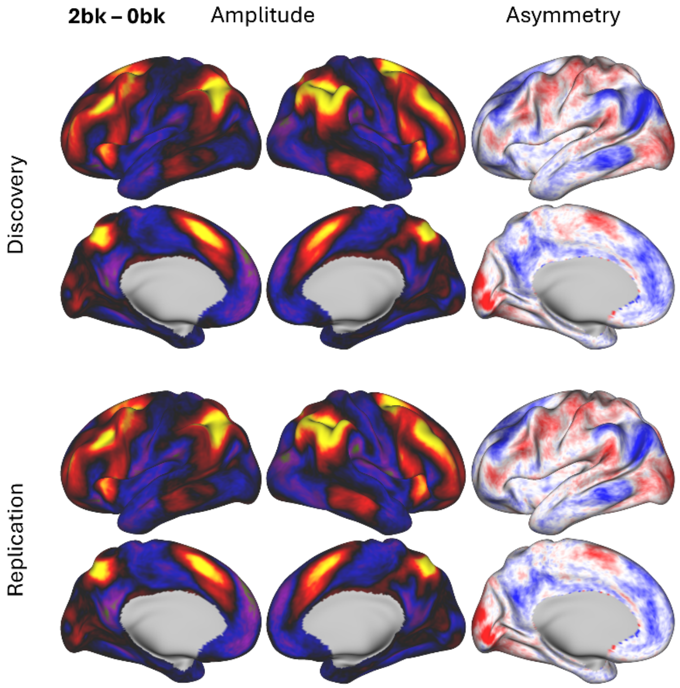

Figure S2. Reproducibility of BOLD signal amplitude and asymmetry for the working memory load contrast (2-back>0-back) in Discovery (n=504) and Replication (n=485) subsamples. See also supplementary Statistical results in CIFTI format.

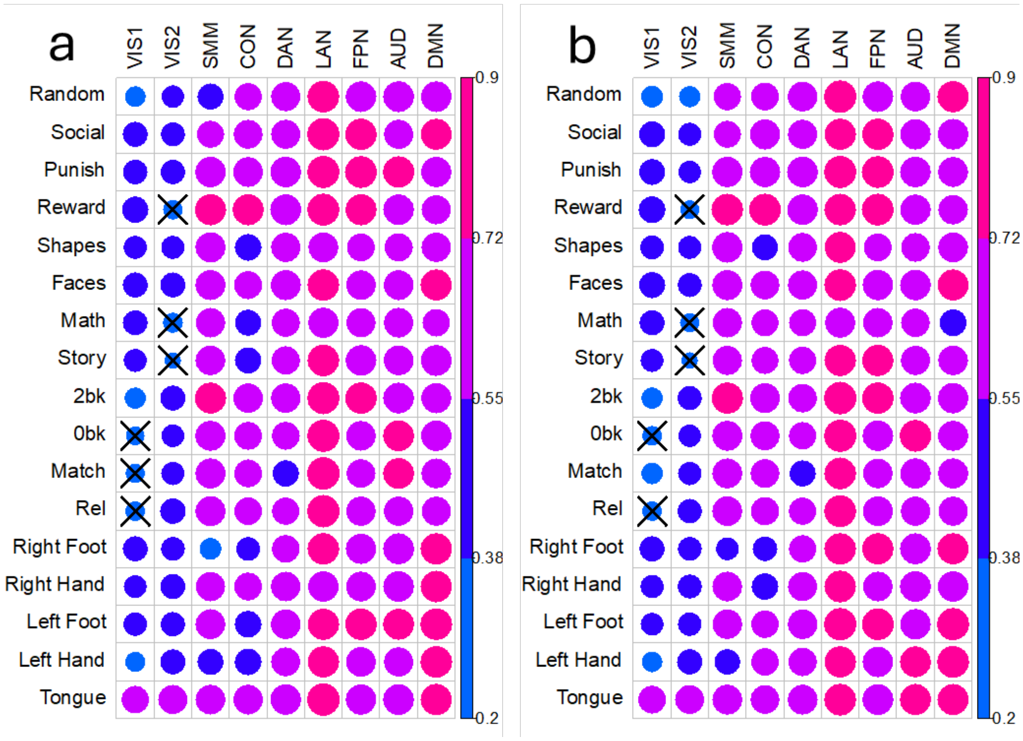

Figure S3. Pearson correlation between BOLD signal amplitude and asymmetry in the left (a) and right (b) cortical hemispheres across 989 healthy adults for 17 task epochs and 9 major networks: Visual (VIS1 and VIS2), Somatomotor (SMM), Cingulo-Opercular (CON), Dorsal Attention (DAN), Language (LAN), Frontoparietal (FPN), Auditory (AUD), Default-Mode (DMN). Non-significant correlations ($p \geq 0.001$) are marked with a black cross. The color of each circle represents the direction and strength of the correlation, while the area of each circle is proportional to the absolute value of the correlation coefficient, reflecting its magnitude.

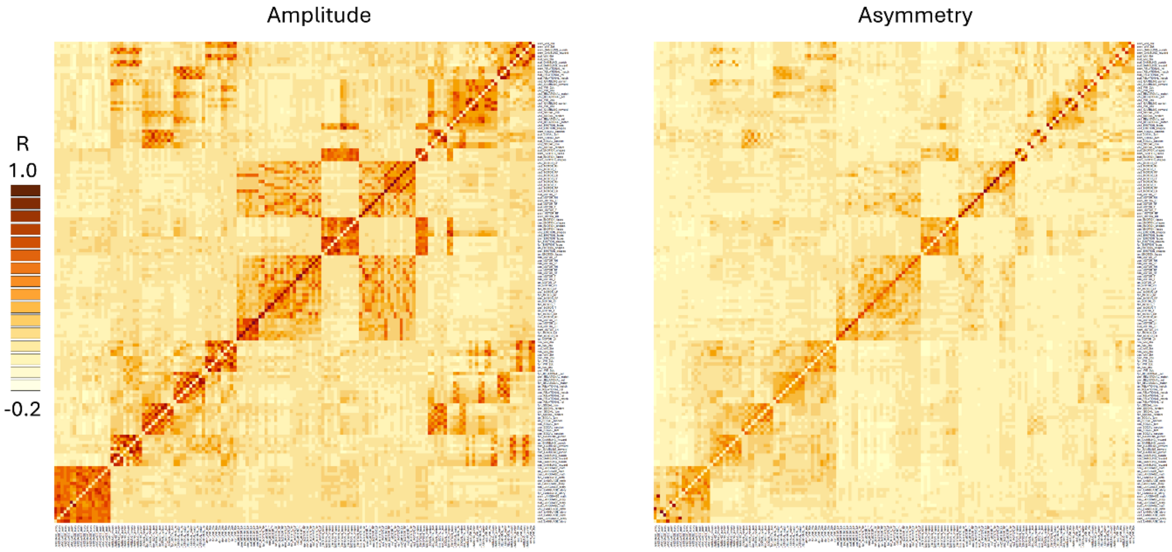

Figure S4. Correlation matrices of BOLD signal amplitude (left) and asymmetry (right) across 9 networks and 17 task epochs (n=989).

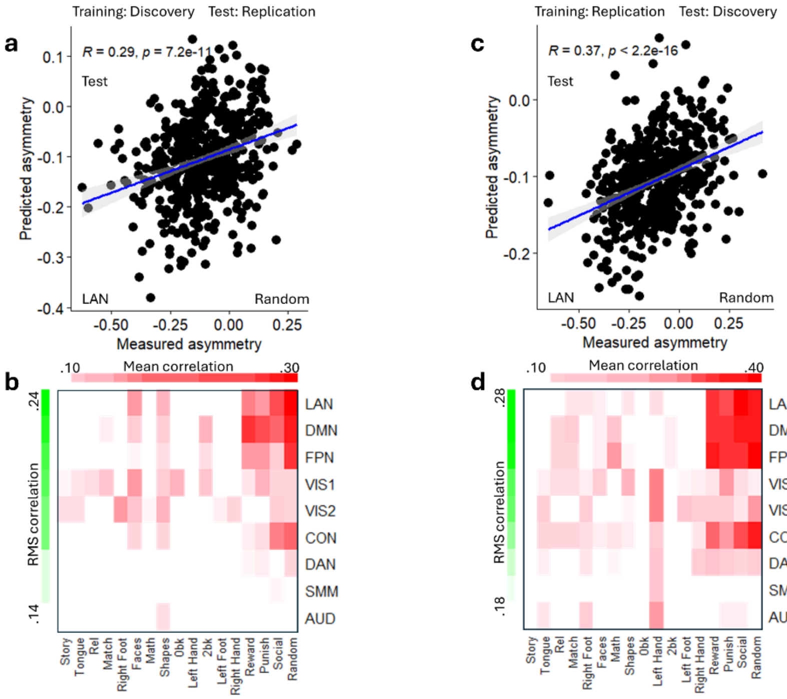

Figure S5. 2-fold cross-validation of partial least square (PLS) prediction of asymmetry. Scatter plots showing linear associations between measured and predicted accuracy for the language network (LAN) and the Random epoch of the social task, based on average amplitudes from 17 BOLD-fMRI contrasts across 9 major networks. The Discovery subsample (n=504) was used to train the PLS regression model with 10-fold cross-validation and 12 PLS components, and the Replication subsample (n=485) was used to test the optimal model; **a-b**). The reversed process—training the PLS model in the Replication sample and using it to predict

asymmetry from amplitude in the Discovery sample—was used to complete the cross-validation (**c-d**). Network abbreviations: VIS1 and VIS2, visual; CON, cingulo-opercular, DAN, dorsal-attention; SMM, somatomotor; FPN, frontoparietal; AUD, auditory; DMN, default-mode; PMM and VMM, Posterior and Ventral Multimodal; ORA, orbito-affective. P<0.05, uncorrected.

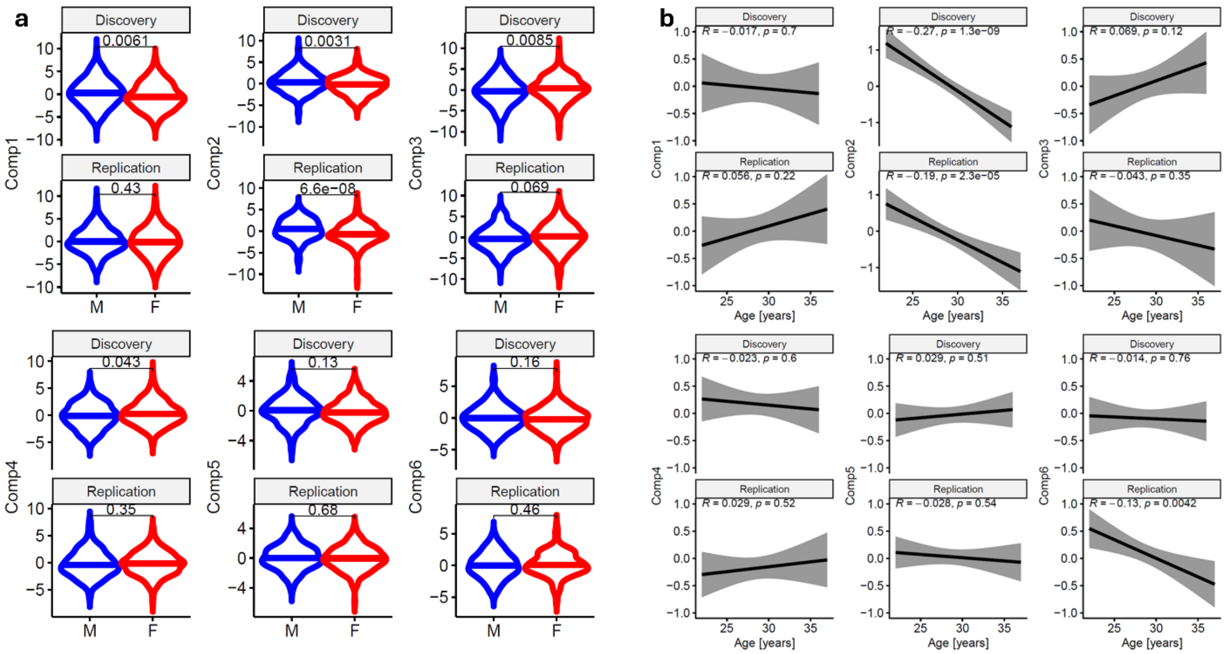

Figure S6. Reproducibility of the effects of sex (**a**) and age (**b**) on the scores of the first 6 components of the prediction of accuracy from BOLD signal amplitude using partial least square (PLS) repression (n=989).

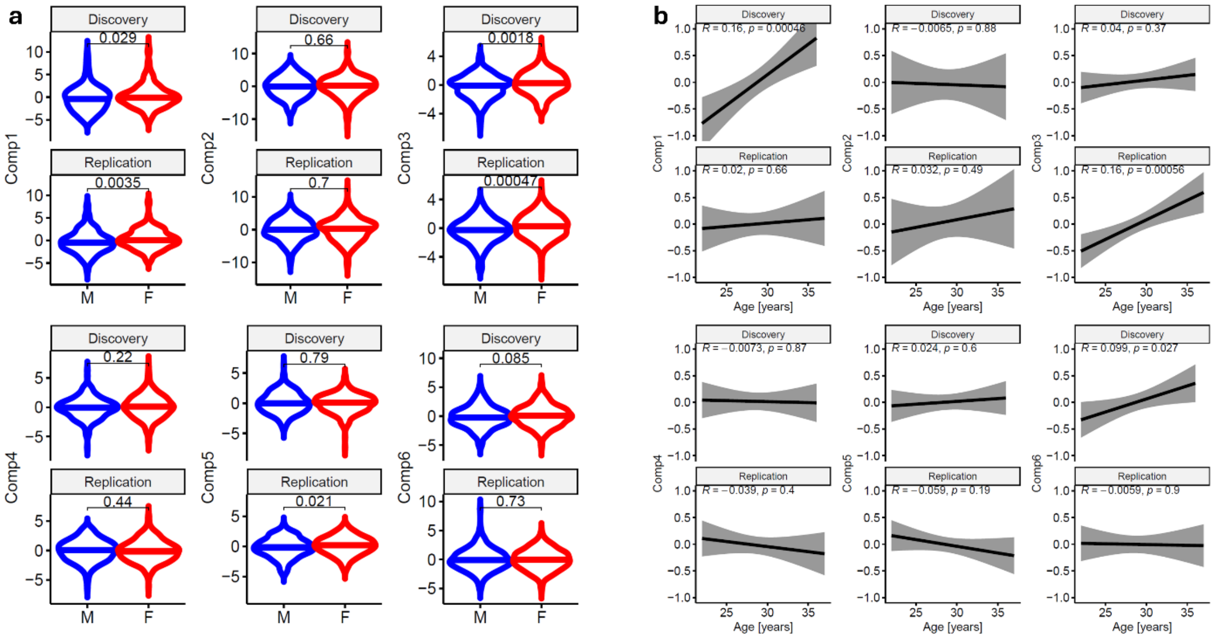

Figure S7. Reproducibility of the effects of sex (**a**) and age **(b**) on the scores of the first 6 components of the prediction of accuracy from asymmetry using partial least square (PLS) repression (n=989).

Supplementary Tables

Table S1: Demographic variables and task accuracy for Discovery and Replication subsamples

Variable/Task epoch	Unit	Discovery	Replication	P-value
Male/Female		237/267	232/253	0.85†
Age	years	28.8(3.7)	28.7(3.7)	0.68*
Black		65	72	
American Indian/Alaskan		34	29	
White		380	370	
Other		25	14	0.25†
BMI	kg/m^2	26.2(5.0)	26.6(5.1)	0.27*
Left handers		53	37	0.13
Accuracy:				
Faces	%	98.2(3.9)	98.5(3.5)	0.34*
Shapes	%	96.7(4.6)	96.7(4.5)	0.90*
Reward	%	50.4(15.0)	49.4(16.1)	0.27*
Punish	%	51.3(11.9)	49.9(13.8)	0.10*
Story	%	94.4(8.2)	94.1(8.6)	0.63*
Math	%	84.0(9.7)	83.8(10.0)	0.70*
Rel	%	66.0(18.7)	65.4(17.8)	0.56*

**Match**	%	87.0(11.6)	87.0(12.2)	0.95*
**Social**	%	49.2(8.7)	49.1(8.4)	0.86*
**Random**	%	44.1(11.1)	44.0(10.1)	0.92*
**0-back**	%	90.2(10.5)	90.3(10.4)	0.84*
**2-back**	%	83.7(10.7)	84.1(10.4)	0.53*

Statistics from χ^2 test (Ŧ) or t-test (*).
