# Supplementary material for: Associations between fMRI signal amplitude, hemispheric asymmetry, and task performance
Source: Commun Biol. 2025 Oct 8;8:1439. doi: 10.1038/s42003-025-08843-w (PMC12508031; doi:10.1038/s42003-025-08843-w)
Supplement: Supplementary file 2 — Reporting Summary [file 42003_2025_8843_MOESM2_ESM.pdf]

nature portfolio

Corresponding author(s): Dardo Tomasi

Last updated by author(s): Jul 1, 2025

Reporting Summary

Nature Portfolio wishes to improve the reproducibility of the work that we publish. This form provides structure for consistency and transparency in reporting. For further information on Nature Portfolio policies, see our Editorial Policies and the Editorial Policy Checklist.

Statistics

For all statistical analyses, confirm that the following items are present in the figure legend, table legend, main text, or Methods section.

n/a	Confirmed	
☐	☒	The exact sample size (*n*) for each experimental group/condition, given as a discrete number and unit of measurement
☐	☒	A statement on whether measurements were taken from distinct samples or whether the same sample was measured repeatedly
☐	☒	The statistical test(s) used AND whether they are one- or two-sided<br>*Only common tests should be described solely by name; describe more complex techniques in the Methods section.*
☐	☒	A description of all covariates tested
☐	☒	A description of any assumptions or corrections, such as tests of normality and adjustment for multiple comparisons
☐	☒	A full description of the statistical parameters including central tendency (e.g. means) or other basic estimates (e.g. regression coefficient) AND variation (e.g. standard deviation) or associated estimates of uncertainty (e.g. confidence intervals)
☐	☒	For null hypothesis testing, the test statistic (e.g. *F*, *t*, *r*) with confidence intervals, effect sizes, degrees of freedom and *P* value noted<br>*Give P values as exact values whenever suitable.*
☒	☐	For Bayesian analysis, information on the choice of priors and Markov chain Monte Carlo settings
☒	☐	For hierarchical and complex designs, identification of the appropriate level for tests and full reporting of outcomes
☐	☒	Estimates of effect sizes (e.g. Cohen's *d*, Pearson's *r*), indicating how they were calculated

Our web collection on statistics for biologists contains articles on many of the points above.

Software and code

Policy information about availability of computer code

Data collection	The data for this study was collected by the Human Connectome Project (HCP). However, the consortium's data collection procedures do not disclose the specific software tools and code implementations used in data acquisition and processing to external parties. As a result, we do not have access to detailed information regarding the software used in the HCP study.
Data analysis	MATLAB R2023a; R4.0; RStudio 2023.03.0

For manuscripts utilizing custom algorithms or software that are central to the research but not yet described in published literature, software must be made available to editors and reviewers. We strongly encourage code deposition in a community repository (e.g. GitHub). See the Nature Portfolio guidelines for submitting code & software for further information.

Data

Policy information about availability of data

All manuscripts must include a data availability statement. This statement should provide the following information, where applicable:

- Accession codes, unique identifiers, or web links for publicly available datasets
- A description of any restrictions on data availability
- For clinical datasets or third party data, please ensure that the statement adheres to our policy

HCP data is publicly accessible through the ConnectomeDB data management platform (https://db.humanconnectome.org/)

Research involving human participants, their data, or biological material

Policy information about studies with human participants or human data. See also policy information about sex, gender (identity/presentation), and sexual orientation and race, ethnicity and racism.

Reporting on sex and gender	This study identifies differences in brain asymmetry between males and females. The HCP study, which provided the data for this research, collected both sex information, ensuring thorough data collection practices. Informed consent for the sharing of individual-level data was obtained by the HCP study, in compliance with ethical standards and privacy regulations.
Reporting on race, ethnicity, or other socially relevant groupings	Participants from a wide range of racial backgrounds were included in the HCP study, ensuring representation across various racial groups. Racial classification was based on self-report, allowing participants to identify their race according to their own understanding. Due to the limited sample size, we could not comprehensively assess the specific effects of race on brain asymmetry. However, race information was used as a covariate in the statistical analyses to reduce potential confounding effects on brain asymmetry.
Population characteristics	The HCP Young Adult cohort consists of approximately 1,200 healthy individuals aged 22 to 37 years, selected to represent a neurologically and psychiatrically healthy population. The cohort is balanced by gender, with roughly equal representation of males and females, and predominantly composed of individuals identifying as European-American (~80-85%), with smaller representations of other racial and ethnic groups such as African-American, Asian, and Hispanic participants. Most participants are right-handed, although the cohort includes a subset of left-handed individuals to support investigations of brain asymmetry. Participants were screened for general health, with exclusions for major psychiatric or neurological disorders, severe medical conditions, or use of psychoactive drugs, ensuring a focus on normative brain function and structure. The sample reflects a cross-section of young adults with varied educational and socioeconomic backgrounds, offering a comprehensive resource for studying brain-behavior relationships in healthy adults.
Recruitment	Subjects were recruited from the United States with an emphasis on ensuring a diverse and healthy population aged 22 to 37 years. Recruitment strategies included outreach to local communities, universities, and advertisements to capture individuals across a range of educational and socioeconomic backgrounds. Participants were carefully screened to exclude individuals with significant psychiatric, neurological, or severe medical conditions, as well as those using psychoactive drugs, ensuring a focus on normative brain function. In addition, efforts were made to include both right-handed and left-handed individuals, although right-handed participants were the majority. All participants provided informed consent and underwent rigorous assessments, including cognitive, behavioral, and imaging measures, as part of the project's goal to create a high-quality, publicly available dataset for mapping brain connectivity in healthy young adults.
Ethics oversight	The HCP Young Adult cohort adhered to strict ethical guidelines to ensure the protection and well-being of its participants. The study received approval from the Institutional Review Board (IRB) at Washington University in St. Louis, which oversaw all aspects of the research. Participants provided written informed consent after being fully briefed on the study's goals, procedures, and potential risks. Special care was taken to maintain participant confidentiality, with all data de-identified before public release. The study complied with the principles outlined in the Declaration of Helsinki, emphasizing respect for participants' autonomy and the minimization of harm. Regular monitoring by the IRB ensured continued compliance with ethical standards throughout the data collection process.

Note that full information on the approval of the study protocol must also be provided in the manuscript.

Field-specific reporting

Please select the one below that is the best fit for your research. If you are not sure, read the appropriate sections before making your selection.

☒ Life sciences ☐ Behavioural & social sciences ☐ Ecological, evolutionary & environmental sciences

For a reference copy of the document with all sections, see nature.com/documents/nr-reporting-summary-flat.pdf

Life sciences study design

All studies must disclose on these points even when the disclosure is negative.

Sample size	Nine-hundred and eigthy-nine participants were included.
Data exclusions	One-hundred and sixty-one participants were excluded from the study due to incomplete image datasets
Replication	Participants were half split into 2 independent demographically matched subsamples (Discovery and Replication). There were no significant differences in brain volume, age, head motion, and race/ethnicity groups between the Discovery and Replication subsamples. All attempts to replication were successful.
Randomization	We used a sample randomization method, where participants were randomly assigned to Discovery and Replication subsamples.
Blinding	In this study, blinding was not implemented due to the nature of the research and its objectives.

Reporting for specific materials, systems and methods

We require information from authors about some types of materials, experimental systems and methods used in many studies. Here, indicate whether each material, system or method listed is relevant to your study. If you are not sure if a list item applies to your research, read the appropriate section before selecting a response.

Materials & experimental systems

n/a	Involved in the study
☒	☐ Antibodies
☒	☐ Eukaryotic cell lines
☒	☐ Palaeontology and archaeology
☒	☐ Animals and other organisms
☐	☒ Clinical data
☒	☐ Dual use research of concern
☒	☐ Plants

Methods

n/a	Involved in the study
☒	☐ ChIP-seq
☒	☐ Flow cytometry
☐	☒ MRI-based neuroimaging

Clinical data

Policy information about clinical studies

All manuscripts should comply with the ICMJE guidelines for publication of clinical research and a completed CONSORT checklist must be included with all submissions.

Clinical trial registration	the HCP is primarily an observational study focused on mapping human brain connectivity in healthy adults, rather than testing medical interventions.
Study protocol	https://www.humanconnectome.org/hcp-protocols-ya-task-fmri
Data collection	HCP data collection was designed to comprehensively map human brain connectivity using advanced neuroimaging and behavioral assessments. Participants underwent multimodal magnetic resonance imaging (MRI) sessions, including structural MRI, diffusion MRI (dMRI), and resting-state and task-based functional MRI (fMRI), acquired on customized scanners optimized for high spatial and temporal resolution. These imaging modalities were complemented by magnetoencephalography (MEG) and electroencephalography (EEG) data in some cohorts. In addition to imaging, participants completed extensive behavioral and cognitive assessments to explore brain-behavior relationships, covering domains such as memory, attention, and executive function. Data collection adhered to standardized protocols to ensure consistency and quality across all participants, with rigorous quality control measures applied to maximize the reliability and usability of the dataset. The result is one of the most comprehensive and high-resolution datasets for studying human brain structure, function, and connectivity.
Outcomes	The HCP has significantly advanced our understanding of human brain structure, function, and connectivity. By providing a detailed map of brain networks, the HCP has facilitated the identification of individual variability in brain connectivity patterns and their relationships with cognition, behavior, and genetics. Key findings include the discovery of distinct brain networks responsible for different cognitive processes, such as attention, memory, and decision-making, as well as insights into the dynamic changes in brain connectivity across the lifespan. The HCP has also enabled the exploration of the genetic and environmental factors influencing brain structure and function. In addition, its vast, publicly accessible dataset has served as a valuable resource for researchers worldwide, promoting the development of new analytical techniques and enhancing studies in various fields, including neurodevelopment, aging, and neuropsychiatric disorders. Ultimately, the HCP has established a foundational framework for future research on the human brain, offering new avenues for understanding brain health and disease.

Plants

Seed stocks	n/a
Novel plant genotypes	n/a
Authentication	n/a

Magnetic resonance imaging

Experimental design

Design type

The tasks were selected to cover diverse domains such as attention, memory, executive function, and emotional processing, with the aim of mapping brain activity associated with these cognitive processes. Each task was optimized for use in a high-resolution fMRI environment, ensuring that brain activity could be measured with precision. Tasks included well-established paradigms such as the working memory n-back task, the emotional face matching task, and the stop-signal task, among others. These tasks were designed to engage specific neural networks, allowing for the identification of key brain regions involved in cognitive control, decision-making, and emotional processing. The fMRI tasks were also carefully balanced in terms of cognitive load and task duration to minimize participant fatigue, while maximizing the collection of robust and reliable data. Together, the diverse set of tasks provides a comprehensive view of brain function in healthy young adults, supporting investigations into how brain networks support complex cognitive behaviors.

Design specifications

The fMRI tasks used a block design that increases statistical power and is ideal for detecting sustained brain activation across multiple regions.

Behavioral performance measures

Behavioral performance measures for the fMRI tasks in the HCP were designed to assess various aspects of cognitive function alongside brain activity. Key measures include accuracy, which reflects participants' ability to complete tasks correctly, and reaction time (RT), which provides insight into cognitive processing speed. In tasks like the n-back and stop-signal tasks, accuracy and RT were used to assess working memory and inhibitory control, respectively. Error rates, including commission and omission errors, were also tracked to understand cognitive control and attention. Additionally, response consistency was measured to evaluate how reliably participants performed across trials. In some tasks, participants provided subjective difficulty ratings to contextualize their performance, and task performance variability was assessed to examine fluctuations in attention and cognitive load. Together, these behavioral measures offer a comprehensive view of participants' cognitive abilities, enabling researchers to explore the relationship between brain function and behavior across a variety of cognitive domains.

Acquisition

Imaging type(s)

functional and structural

Field strength

3T

Sequence & imaging parameters

3D Tlw inversion prepared RF-spoiled gradient echo and T2w variable flip angle fast spin echo pulse sequences with 0.8 mm isotropic resolution were used for structural MRI. T2*-weighted multiband EPI (TE/ TR=33/720 ms, 2 mm isotropic resolution, 72 slices covering the entire brain, slice acceleration=8, and flip angle=52 degree) was used to acquire functional MRI (fMRI) data with blood-oxygen-level-dependent contrast.

Area of acquisition

Whole brain acquisition was used.

Diffusion MRI ☐ Used ☒ Not used

Preprocessing

Preprocessing software

Freesurfer (v5.3.0), FSL(v5.0.6), HCP pipeline.

Normalization

Spatial normalization of fMRI data was performed using a minimally processed pipeline designed to preserve the high spatial resolution of the data while aligning it to a common reference space. This process involves co-registering each individual's fMRI data to their high-resolution structural T1-weighted images, followed by transformation into the MNI (Montreal Neurological Institute) space. The minimally processed pipeline ensures that the fMRI data retains its native spatial properties, allowing for accurate comparisons across subjects while minimizing distortions from the normalization process. This approach supports the integration of fMRI data with other modalities.

Normalization template

MNI152

Noise and artifact removal

Artifacts, such as those caused by head motion, cardiac, and respiratory fluctuations, are carefully addressed to enhance the quality of the task fMRI data. The HCP preprocessing pipeline includes several steps to mitigate these artifacts. First, motion correction is performed to align all functional images within a session, minimizing the effects of head movement during scanning. To address physiological noise, retrospective motion regression and physiological noise modeling are used. The pipeline incorporates information from physiological recordings (e.g., heart rate and respiration) obtained during scanning to model and remove these sources of noise. Temporal filtering is applied to reduce low-frequency drifts and high-frequency noise.

Volume censoring

The minimally preprocessed HCP pipelines do not include censoring as part of their standard processing steps, as the goal is to remove as little data as possible and preserve the raw signal.

Statistical modeling & inference

Model type and settings

Two-sided t-test

Effect(s) tested: Functional asymmetry for 28 task-fMRI contrasts.

Specify type of analysis: ☐ Whole brain ☐ ROI-based ☒ Both

Anatomical location(s): 12 network partitions, 360 cortical ROIs, and 19 subcortical ROIs.

Statistic type for inference (See Eklund et al. 2016): vertex-wise and ROI-wise

Correction: FDR

Models & analysis

n/a	Involved in the study
☐	☒ Functional and/or effective connectivity
☒	☐ Graph analysis
☐	☒ Multivariate modeling or predictive analysis

Functional and/or effective connectivity: n/a

Multivariate modeling and predictive analysis: Partial Least Squares (PLS) multivariate modeling was employed to predict functional asymmetry and task accuracy based on BOLD signal amplitude. This approach enabled the exploration of how variations in brain activity, measured through BOLD signal amplitude during task performance, relate to individual differences in functional brain asymmetry and cognitive performance. By using PLS, the model identified latent variables that captured the joint patterns of brain activation and behavioral outcomes, offering a comprehensive understanding of how brain function supports cognitive processes. The analysis specifically examined how asymmetries in brain activity across different regions and networks could predict task accuracy, providing insights into the neural mechanisms that underlie performance variations across individuals. This approach facilitated a data-driven way to uncover relationships between brain function and behavior without predefined hypotheses about the specific neural substrates involved.
